# Selection of Inhibitor-Resistant Viral Potassium Channels Identifies a Selectivity Filter Site that Affects Barium and Amantadine Block

**DOI:** 10.1371/journal.pone.0007496

**Published:** 2009-10-16

**Authors:** Franck C. Chatelain, Sabrina Gazzarrini, Yuichiro Fujiwara, Cristina Arrigoni, Courtney Domigan, Giuseppina Ferrara, Carlos Pantoja, Gerhard Thiel, Anna Moroni, Daniel L. Minor

**Affiliations:** 1 Cardiovascular Research Institute, University of California San Francisco, San Francisco, California, United States of America; 2 Dipartimento di Biologia e Istituto di Biofisica del Consiglio Nazionale delle Ricerche, Università degli Studi di Milano, Milan, Italy; 3 Department of Biochemistry and Biophysics, University of California San Francisco, San Francisco, California, United States of America; 4 Department of Cellular and Molecular Pharmacology, University of California San Francisco, San Francisco, California, United States of America; 5 Department of California Institute for Quantitative Biomedical Research, University of California San Francisco, San Francisco, California, United States of America; 6 Technische Universität Darmstadt, Institute für Botanik, Darmstadt, Germany; 7 Physical Biosciences Division, Lawrence Berkeley National Laboratory, Berkeley, California, United States of America; Yale School of Medicine, United States of America

## Abstract

**Background:**

Understanding the interactions between ion channels and blockers remains an important goal that has implications for delineating the basic mechanisms of ion channel function and for the discovery and development of ion channel directed drugs.

**Methodology/Principal Findings:**

We used genetic selection methods to probe the interaction of two ion channel blockers, barium and amantadine, with the miniature viral potassium channel Kcv. Selection for Kcv mutants that were resistant to either blocker identified a mutant bearing multiple changes that was resistant to both. Implementation of a PCR shuffling and backcrossing procedure uncovered that the blocker resistance could be attributed to a single change, T63S, at a position that is likely to form the binding site for the inner ion in the selectivity filter (site 4). A combination of electrophysiological and biochemical assays revealed a distinct difference in the ability of the mutant channel to interact with the blockers. Studies of the analogous mutation in the mammalian inward rectifier Kir2.1 show that the T→S mutation affects barium block as well as the stability of the conductive state. Comparison of the effects of similar barium resistant mutations in Kcv and Kir2.1 shows that neighboring amino acids in the Kcv selectivity filter affect blocker binding.

**Conclusions/Significance:**

The data support the idea that permeant ions have an integral role in stabilizing potassium channel structure, suggest that both barium and amantadine act at a similar site, and demonstrate how genetic selections can be used to map blocker binding sites and reveal mechanistic features.

## Introduction

Ion channels produce the bioelectrical signals that drive sensory signals, locomotion, cognition, and in some cases viral infection [1]. Because of the diversity of functions and ion channel types there is a great interest in developing methods to identify and analyze modifiers of ion channel function [2]–[5]. As membrane proteins, ion channels remain a challenging target for high throughput approaches [2], [3], [6]. Thus, the development of new approaches to identify and map the sites of action of agents that can be used to control channel function remains an important goal. Genetic selections provide a potentially powerful means to identify and uncover the mechanisms of action of ion channel modifiers. Such approaches have the additional advantage of finding compounds that directly affect function [4]. To date, a number of efforts have explored the potential for using ion channel directed selections in yeast (*S. cerevisiae*) [7]–[9] and worms (*C. elegans*) [10]–[12] as a way to identify novel ion channel blockers. Beyond modifier identification, the use of genetic selection systems to identify modifier-resistant channels has been shown to provide a facile and unbiased means to identify residues that are likely to be involved in the mechanism of action [9], [10].

**Figure 1:**
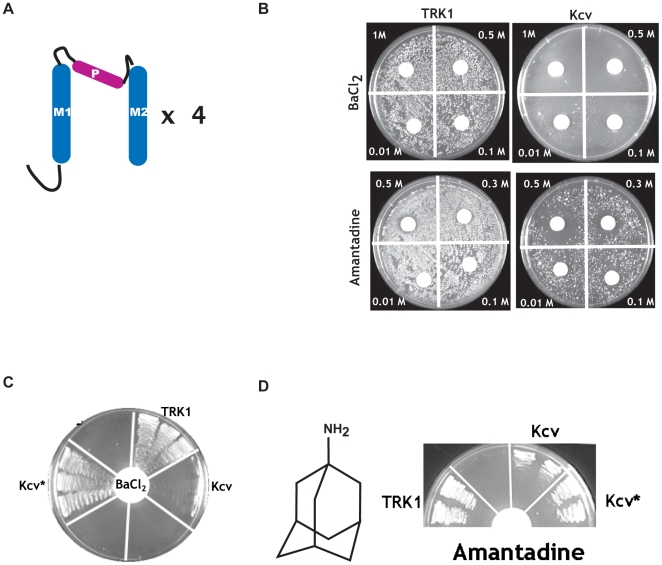
Blockers affect Kcv-dependent rescue of potassium transport deficient yeast. A, Cartoon showing the Kcv topology. The two transmembrane segments (M1 and M2) and the pore-forming region (P) are indicated. Four monomers assemble to make a functional channel. B, Examples of the effects of blockers applied at the indicated concentrations to yeast grown on selective media containing 0.5 mM KCl. Yeast express the transporter TRK1 and Kcv, as indicated. TRK1 image for barium is identical to [9]. C, Barium-resistant rescue of growth by the mutant Kcv* on 0.5 mM KCl media in the presence of 10 mM BaCl_2_. D, Amantadine-resistant rescue of growth by the mutant Kcv* on 0.5 mM KCl media in the presence of 500 mM amantadine. Chemical structure of amantadine is shown (left).

Kcv is the smallest potassium channel known to be expressed naturally in a eukaryotic cell and is part of a family of channels present in a variety of viral sources [13]. A single Kcv subunit is composed of 94 amino acids that encode a short N-terminal helix and two transmembrane segments that are bridged by a pore forming region that contains the hallmark potassium channel selectivity filter (Figure 1A). As with many other potassium channels, Kcv forms tetramers [14], [15]. Because of its minimal size, Kcv is an excellent model system for probing ion channel structure-function relationships. Here, we report the expansion of the yeast screening method to understand the action of an ionic blocker, barium, and a small molecule blocker, amantadine, that affects the function of Kcv as well as other viral channels [16]–[18]. Our results demonstrate the utility of combining blocker screening and selection of blocker resistant mutants as a means to probe how channels and blockers interact.

## Results

### Selection of Kcv mutants resistant to barium and amantadine

Rescue of the growth of potassium transport deficient yeast strains by functional ion channels provides a means for testing structure-function relationships regarding both channel activity [19]–[26] and interaction with blockers [7]–[9]. In an effort to expand the number of functional targets that can be studied using the *Δtrk1Δtrk2* potassium transport deficient yeast system [27], we found that heterologous expression of the viral channel Kcv was able to complement growth under low potassium conditions [28]. Further studies using a plate assay in which blocking compounds are applied via Whatman filter discs [9] indicated that both barium and amantadine, two reagents that block Kcv function [16], prevented Kcv-mediated rescue but did not affect the ability of heterologous expression of the yeast potassium transporter TRK1 to rescue growth (Figure 1B). These observations provide further evidence that Kcv forms functional potassium channels in yeast that can be specifically affected by known channel blockers and gave the opportunity to explore the nature of the channel-blocker interactions using selection methods.

Previous studies have demonstrated that selection for blocker resistant channels can lead to the identification of amino acids playing key roles in blocking mechanisms [9]. To establish whether we could apply such selection methods to Kcv, and as a second means for verifying that the barium and amantadine mediated effects resulted from block of functional Kcv channels, we constructed a Kcv mutant library and subjected the library to selections aimed at identifying blocker-resistant mutants.

Because of the small size of the coding region of the gene (282 bases), we used a mutagenic PCR approach to make the library as it permitted us to generate mutations throughout the entire gene. Sequencing of a set of twelve unselected Kcv mutants showed that the PCR procedure produced mutants bearing one to eight amino acid mutations and an average number of four amino acid changes that were distributed throughout the Kcv gene. Selection experiments in the absence of any blockers indicated that ∼25% of the mutants in the library were functional under selective conditions (1 mM KCl) and a smaller fraction ∼14% were functional under the most stringent selection conditions (0.5 mM KCl). These results indicate that even though many of the mutant channels bear multiple changes, a reasonable fraction of the mutants maintain some level of function. We then used this library to search for Kcv mutants that would be resistant to either blocker by conducting selections for Kcv mutants that could rescue the potassium-deficient yeast strain in the presence of barium or amantadine under conditions where channel function was required for survival.

For these selections, the Kcv library was transformed into potassium transport deficient yeast and plated on permissive media under conditions in which the channel function was not required for survival. Established colonies were replica plated onto low potassium plates and challenged with different concentrations of blocker applied via Whatman filters. Picking colonies that grew near the filters in the zone of highest blocker concentration and retesting for the phenotype identified five barium resistant mutants and three amantadine resistant mutants (Table 1). Intriguingly, one mutant, termed Kcv*, was found in both selections. This mutant bore eight amino acid changes (K6R, E12G, K47T, T63S, T74A, H83Q, T86S, V87A) and because it was resistant to both barium and amantadine (Figure 1C and D) was chosen for further study.

**Table 1:** Results of selection for blocker-resistant Kcv mutants.

Selection condition	Blocker resistant clones
1 mM KCl,100 mM Barium	T60A/V87E/L92Q, I20F/K29R/L70Q/F88V, F66L/D98Y/L92P, I20V/N37D/D68G/I84V/L92P
0.5 mM KCl, 10 mM Barium	K6R/E12G/K47T/T63S/T74A/H83Q/T86S/V87A*
1 mM KCl, 500 mM Amantadine	K6R/E12G/K47T/T63S/T74A/H83Q/T86S/V87A*, E12G/F14L/K77E/F88S
0.5 mM KCl, 500 mM Amantadine	K6R/E12G/K47T/T63S/T74A/H83Q/T86S/V87A*

*indicates sequences identified in multiple conditions.

**Figure 2:**
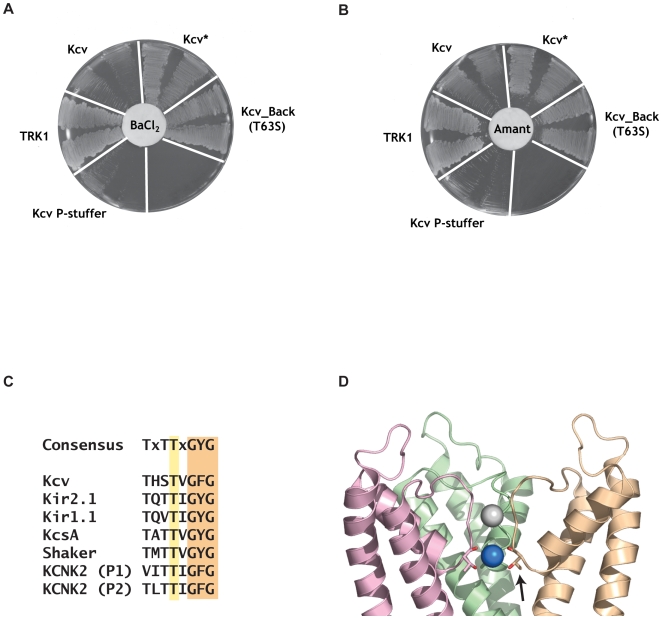
Kcv Blocker-resistant mutants of potassium transport deficient yeast. Rescue in the presence of barium (10 mM BaCl_2_) A, and amantadine (500 mM) B, by the Kcv* and Kcv_Back mutant identified by backcrossing. Kcv_Back contains a single point mutation, T63S. Kcv P-Stuffer is a non-functional Kcv construct (see Materials and Methods). C, Sequence alignment of the selectivity filter regions of Kcv, Kir2.1, and select potassium channels. The positions of Kcv T63 and Kir2.1 T142 are highlighted in yellow. The selectivity filter ‘GYG’ sequence is also highlighted. D, Model indicating the position of the conserved T63 position (shown in sticks and having one of the copies indicated by the arrow) using KcsA [31]. The site 4 ion, which is coordinated by both backbone and sidechain oxygens from the T63 position is shown as a blue sphere. The front subunit of the KcsA tetramer is not shown.

Because Kcv* bore eight amino acid changes, we used a PCR backcrossing procedure [29] as a way to dilute the mutations into a wild-type background to address which mutations were necessary for the phenotype and probe whether multiple changes working in concert were required for the apparent blocker resistance. Selection of barium and amantadine-resistant mutants from this procedure uncovered a mutant, Kcv_Back, that was found to bear a single change, T63S, that was responsible for both phenotypes (Figure 2A and B). T63 is part of the hallmark sequence of the potassium channel selectivity filter (Figure 2C) [30]. This element forms the active site of the channel, is the most conserved feature among potassium channels, and makes intimate contacts with the permeant ions [31]. Crystallographic studies have provided structural insight into the nature of potassium channel binding sites for barium [32], [33]. Notably, Kcv T63 occupies a position in the channel selectivity filter that provides a coordination of barium at site 4, the innermost site of the selectivity filter (Fig. 2D). Of all of the hallmark residues, the threonine corresponding to Kcv T63 is the only residue in which the amino acid sidechain coordinates the ion in the filter. Although it seems reasonable to expect that a change at this sidechain position might affect barium block, we were surprised that the subtle change of T→S, which does not remove the coordinating sidechain oxygen, would be sufficient to impact blocker sensitivity.

### Characterization of blocker resistant Kcv mutants

**Figure 3:**
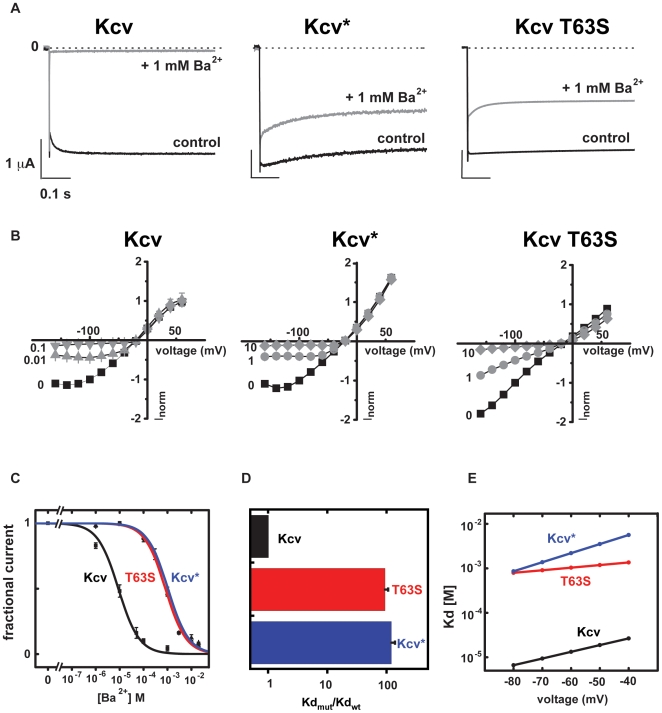
Functional characterization of Kcv mutants. A, Two electrode voltage clamp recording of Kcv, Kcv*, and Kcv T63S in 50 mM external KCl (control) and external KCl with 1 mM Ba^2+^. Test voltage is −60 mV from a holding potential of −20 mV. B, Current-voltage relations for Kcv, Kcv*, and Kcv T63S in the presence of different concentrations of external barium. Currents are normalized to the value at −100 mV for the barium-free solution. External barium concentrations (in mM) are indicated next to each I-V curve. C, Comparison of the relative barium affinities for Kcv (black), Kcv* (blue), and Kcv T63S (red) at −80 mV. Lines indicate fits to the Hill equation I_f_ = Kd^n^/(Kd^n^+[Ba^2+^]^n^). D, Comparison of the relative barium dissociation constants at −80 mV. E, Voltage dependence of steady-state barium block. Lines show fits to the Woodhull equation [63]. Kd(V) = Kd(0)exp(zFδ/RT x V), Kd(0) and δ values are: 0.09±0.48 mM, 28.0±0.9 mM, and 2.3±0.2 mM; δ = 0.33±0.04, 0.53±0.02 and 0.17±0.02 for Kcv, Kcv*, and T63S, respectively. Errors are s.d.

**Figure 4:**
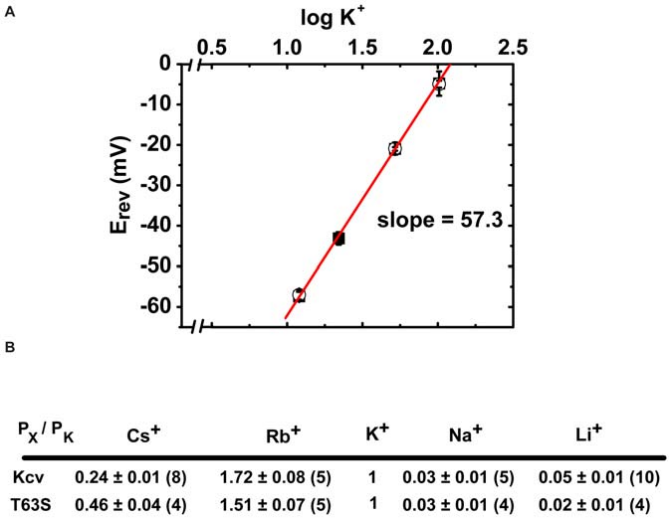
Selectivity to K^+^ and relative permeability to monovalent cations of Kcv and T63S. A, Potassium selectivity of Kcv and Kcv T63S channels. Mean values±s.e. (n = 3) for the current reversal potentials (E_rev_) of Kcv (○) and T63S (•)obtained in 10, 20, 50 and 100 mM external K^+^ are plotted as a function of log K^+^ concentration ([K^+^]_out_). Line represents the theoretical Nernst equation E_K_ = RT/zF ln [K^+^]_out_/[K^+^]_in_ with a slope of 59.2 mV, assuming that in oocytes [K^+^]_in_ = 108.6 mM [64]. B, Permeability of wt Kcv and T63S mutant to different univalent cations. All cations were tested at 50 mM as chloride salts. P_X_/P _K+_ is the permeability of the ion X^+^ relative to K^+^, calculated from current reversal potentials (see Material and Methods). Values are the means±s.e.; number of tested oocytes is indicated in brackets. Values for wt Kcv are from [65].

To determine the electrophysiological consequences of the Kcv mutants, we examined their functional properties in *Xenopus* oocytes using two-electrode voltage-clamp methods. Both Kcv* and T63S make functional ion channels (Figure 3A). There is an apparent change in the rectification properties of T63S relative to wild-type and Kcv* that may be the result of changes in fast gating [34]. In accord with the yeast results, both Kcv* and T63S were found to be resistant to barium block having apparent Kd values that were altered by ∼100 fold (Figure 3C-E). The barium resistance of both mutant channels was similar (Kd_−80 mV_ = 8.8±0.9 µM, wt; 1048±122 µM, Kcv*; 818±68 T63S) and provides further support that the primary source of the barium-resistant phenotype is the single point mutant at T63. Woodhull analysis reveals differences in the voltage dependence of block between Kcv* and T63S that suggest that other mutations in Kcv* may also contribute to the blocker resistant phenotype and alter how the T63S change influences barium-channel interactions. Further characterization of the T63S mutant demonstrated that the T63S change had no effect on the monovalent cation selectivity properties of the channel with respect to sodium, but did reduce permeability to rubidium and lithium and increase the permeability to cesium (Figure 4). Thus, the T63S mutation at the site 4 coordination site changes sensitivity to the divalent blocker sensitivity and also affects how the channel responds to monovalent ions other than sodium.

**Figure 5:**
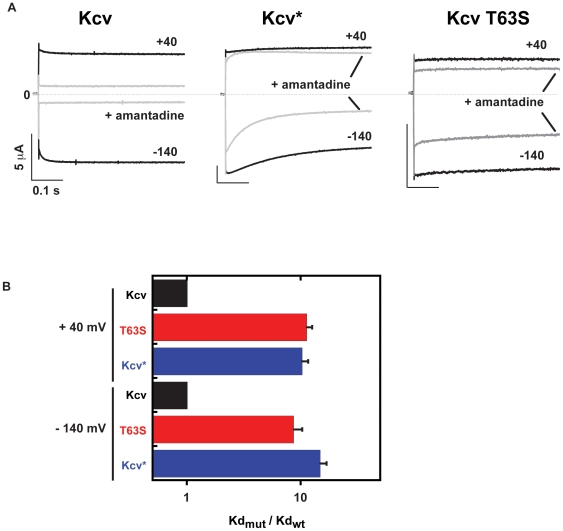
Functional characterization of amantadine sensitivity. A, Current traces at +40 mV and −140 mV in the absence (black) and presence (grey) of 10 mM amantadine for Kcv, Kcv*, and Kcv T63S. B, Comparison of the relative amantadine dissociation constants at +40 mV (2±0.2, wt; 22.4±1.4 Kcv*, 20.5±1.7 T63S) and −140 mV (0.8±0.1, wt; 6.9±0.9 Kcv*, 11.8±0.7 T63S). Errors are s.d.

Wildtype Kcv is blocked by the antiviral compound amantadine (Figure 5A) [16]. Two-electrode voltage clamp studies of amantadine block also showed that both Kcv* and the T63S mutant were substantially less sensitive to the blocker than the wild-type channel (Figure 5). The reduction in amantadine sensitivity by the same mutation that lowers barium sensitivity suggests that even though the two blockers have radically different characters, one being a divalent ion and the other being a small molecule, both act to reduce channel function at a similar site in the filter. Taken together with the functional analysis of barium block, these data provide further support for the utility of the yeast selection as a means to identify key sites that are involved in blocker action.

**Figure 6:**
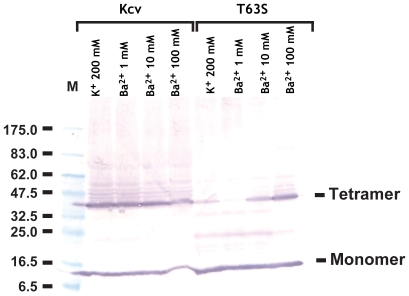
Stability analysis of Kcv and Kcv T63S. Western blot of microsomal membranes containing Kcv or Kcv T63S following treatment with the indicated concentrations of K^+^ or Ba^2+^ prior to loading onto an SDS gel. Positions of the tetramer and monomer forms, and the size of the molecular weight markers are indicated. Additional band in T63S is assigned as the dimer. Channels bear an N-terminal His_9_ tag and were detected using an anti-polyhistidine antibody (see Material and Methods).

Previous studies of purified potassium channels have indicated that the interactions between the ions in the selectivity filter and the channel subunits contributes greatly to the overall stability of the complex [15], [35]–[37]. To characterize further the effect of the T63S mutation on ion binding to the channel, we employed a biochemical assay [15] in which the effects of potassium and barium ions on the stability of the channel tetramer was examined (Figure 6). In both wild-type and T63S channels a high molecular weight band was present in the presence of high barium concentrations. This heavy form of Kcv, which has been assigned as a tetrameric species [15], runs with an anomalous molecular weight that is apparently lower than the expected molecular weight of a tetramer (56 kD), a property that is common to the behavior of many membrane proteins on SDS gels [38], [39] including tetrameric forms of potassium channels [40], [41]. The presence of potassium provided less stabilization of the tetrameric form of the T63S mutant than to the wild-type. Examination of the concentration dependence in the presence of barium showed that the ability of barium to stabilize the tetrameric form was also diminished in the T63S mutant relative to the wild type. Together, these data provide further support for the hypothesis that the T63S mutation affects the ability of the channel to interact with ion that can bind to selectivity filter sites and are consistent with the idea that T63 contributes to the ion interactions at site 4.

### Analysis of the Kcv T63S equivalent mutation in Kir2.1

**Figure 7:**
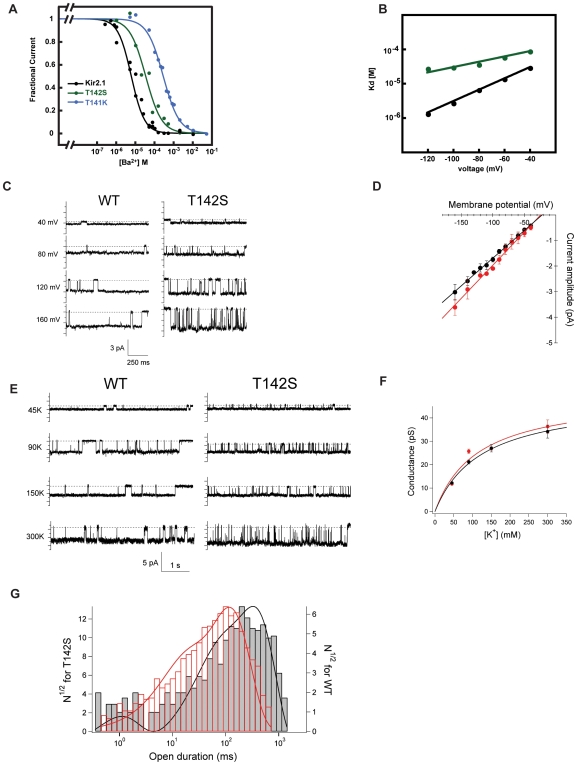
Kir2.1 T142S functional analysis. A, Comparison of the barium Kds for Kir2.1, Kir2.1 T142S (green), and Kir2.1 T141K (blue, data from [9]) B, Woodhull comparison for Kir2.1 and Kir2.1 T142S (green). Lines show fits to the Woodhull equation [63]. Kd(V) = Kd(0)exp(zFδ/RT x V). Kd(0) values are: 131±3 µM and 172±22 µM; δ = 0.56±0.02 and 0.22±0.02 for Kir2.1 and Kir2.1 T142S, respectively. C, Representative current traces of Kir2.1 and Kir2.1 T142S in 90 mM KCl. D, Plot of single channel amplitudes versus membrane potential. E, Single channel current traces for Kir2.1 and Kir2.1 T142S as a function of potassium concentration. F, Single channel conductance as a function of [K^+^]. Fits are to the Michelis-Menten equation and yielded values of Km = 123 mM, 120 mM, and γ_max_ = 48.5 pS and 51.3 pS for Kir2.1 and Kir2.1-T142S, respectively. G, Gating kinetics analysis of Kir2.1 (black) and Kir2.1-T142s (red). Mean open times Kir2.1 298.7±292.2 ms (n = 416) and Kir2.1-T142S 93.4±92.6 ms (n = 1890). Intraburst probability was not changed (0.84±0.041 n = 8, 0.88±0.042 n = 8, Kir2.1 and Kir2.1-T142S, respectively). Errors are s.d.

The Kcv T63 position is one of the most highly conserved positions in potassium channel selectivity filters. In order to gain a better insight into how the subtle change of T→S at this position might affect channel function, we studied the equivalent mutant in the mammalian inward rectifier Kir2.1. Examination of the effects of the introduction of the T→S change at Kir2.1 residue 142 resulted in channels that, similar to Kcv, displayed barium resistant behavior. T142S caused ∼5.4 fold reduction at −80 mV relative to wild-type Kir2.1 (Kd_−80 mV_ = 35.6 µM versus 6.5 µM [9], for T142S and wild type, respectively). These changes are substantially smaller than those previously reported for the changes caused by the introduction of a charged residue at the adjacent position near site 4, T141K (Figure 7A) and are in line with the conservative nature of the T→S change. Remarkably, Woodhull analysis revealed that T142S causes a large change in the voltage dependence of block δ = 0.22±0.02 Kd (0)  = 172±22 µM for T142S versus δ = 0.56±0.02 and Kd (0)  = 131±3 µM for wild-type [9] (Figure 7B). Again, this change stands in contrast to that caused by the previously reported values for the introduction of a charge near site 4 by the T141K mutation where δ = 0.47±0.02 and Kd (0)  = 6421±468 µM [9].

Single channel analysis of T142S revealed that the T142S mutation had minimal effects on single channel conductance or sensitivity to permeant ion concentrations (Figure 7C–F) but caused a marked change in the single channel gating properties (Figure 7G). T142S channels showed flickery behavior that is absent from wild-type channels. T142S channels have a mean open time that is ∼3 fold shorter than wild type (289.7±292.2 ms, Kir2.1, 93.4±92.6 ms, T142S) but display no change in intraburst open probability (0.84±0.041, Kir2.1, 0.88±0.042, T142S) suggesting that the mutation destabilizes the conducting conformation of the selectivity filter. This result agrees with the apparent loss in stability of the Kcv T63S tetramer in the presence of potassium. Taken together, these data provide further support for the idea that the integrity of the site 4 ion binding site is crucial for channel function.

### Negative coupling between Kcv S62T and T63S

**Figure 8:**
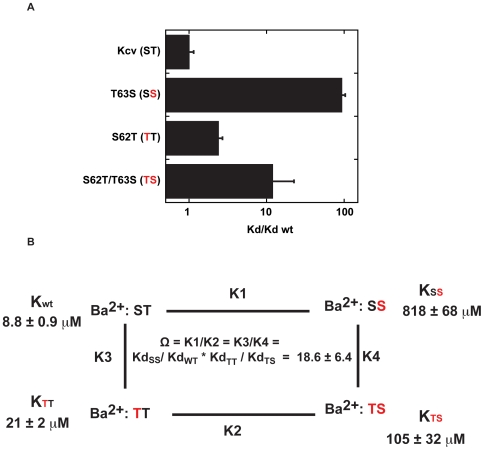
Coupling between Kcv residues 62 and 63 influences barium block. A, Comparison of the relative barium dissociation constants at −80 mV for Kcv, T63S, S62T, and S62T/T63S mutants. Brackets show the two positions under investigation. Red indicates the sites of the mutation. B, Double mutant cycle analysis [42], [43] shows that the changes at positions 62 and 63 are coupled. Ω = 1 for mutations that are energetically independent. Errors are s.d.

Potassium channel selectivity filter sequences are very well conserved [30]. Given this high degree of similarly, it was striking that the T→S change had a much larger impact with respect to apparent barium affinity in Kcv versus Kir2.1 (∼92 fold versus ∼5.4 fold at −80 mV, respectively). Given that starting barium sensitivities were similar (low micromolar at −80 mV) and that the selectivity filter regions are so well conserved, we sought an explanation for the difference. Consideration of the selectivity filter sequences (Figure 2C) shows a notable difference in the Kcv sequence. The residue adjacent to T63 is a serine, whereas the more common residue in potassium channel filters, including that of Kir2.1, is a threonine. To ask whether the presence of the serine at position 62 was responsible for the large impact of the T63S mutation, we introduced a S→T change at residue 62 to make a Kcv sequence, Kcv(TT), that resembles the Kir2.1 filter sequence. This mutation decreased the sensitivity of the channel to barium block by 2.4 fold at −80 mV (Figure 8A). Incorporation of the T63S mutation into this background (Kcv(TS), Figure 8A) reduced sensitivity to barium further and causes a change that when compared with the wild-type is in line with that seen in the Kir2.1 background (∼12 fold relative to wild type). However, this diminution of apparent barium affinity is far smaller than that imparted by T63S alone (Kcv(SS) ∼93 fold) and suggests a negative coupling between the positions with respect to barium binding. Double mutant cycle analysis [42], [43] confirms that there is a strong negative coupling with respect to barium sensitivity caused by the ST→TS double mutation at positions 62 and 63 (Figure 8B). This analysis provides an explanation for why the T63S mutation on its own has a greater impact in Kcv than the equivalent mutation in Kir2.1. The data suggest that the large effect on the barium sensitivity of Kcv by the T→S change at the position 63, the position that can coordinate the site 4 ion, is the absence of the methyl group at the adjacent position.

## Discussion

Understanding the nature of interactions between ion channels and blocking molecules remains an important area of inquiry. The use of genetic selections to identify and examine channel-blocker interactions is a powerful, hypothesis-free means to probe channel-blocker interactions in an unbiased fashion. Recent applications of this approach to both potassium channels [9] and voltage-gated calcium channels [10] has identified a number of new mutations that affect interactions with channel modifiers [4]. Here, by studying the interactions of blockers with the miniature potassium channel Kcv, we identified a single point mutation in a residue that forms intimate contacts with the permeant ions at site 4 in the selectivity filter, T63S, and that affects channel blockade by both the divalent ion barium and the small molecule blocker amantadine.

That the exchange of threonine for serine at site 4 would affect barium binding is somewhat surprising. Previous application of the yeast genetic selection approach to the study of block of the mammalian inward rectifier Kir2.1 yielded a mutation in a residue adjacent to site 4 that does not coordinate the permeant ion but that caused an electrostatic disturbance at site 4. This T→K conferred barium resistance by selectively destabilizing divalent ion binding while sparing interactions with monovalent ions [9], [44]. Unlike the Kir2.1 barium resistant T→K change [9], the T63S mutation retains a sidechain hydroxyl and therefore, does not change the electrostatic environment of the site 4 ion. Further this change should not alter the coordination chemistry as it retains the essential chemical feature of site 4, a hydroxyl that can contribute to the coordination shell of the permeant ion [31] (Figure 2C). It is clear that changes in both sidechain volume and coordination chemistry at this conserved position can affect channel function. A T→C change at the position equivalent to Kcv T63 in KcsA, T74, has been shown to have a devastating effect on both single channel conductance and occupation of site 4 by monovalent ions [45]. We found that the T→S change has similar effects on barium block in the context of two different potassium channels, Kcv and Kir2.1. These data support the idea that the simple change in steric bulk caused by the T→S mutation may be the cause for changes in barium block. Further, in both cases interactions with the natural permeant ion potassium are affected. These are manifested as a loss of channel stability in the presence of potassium for Kcv (Figure 6) and the functional destabilization of the Kir2.1 conducting state (Figure 7C, E, & G). Taken together, the biochemical and the functional data suggest that the mechanism of action of the T→S mutation is a destabilization of the conducting conformation of the selectivity filter. One attractive explanation is that the T→S change impacts the structural dynamics of the filter. This idea is supported by the observation that additional steric bulk at the adjacent position (Kcv 62) mitigates the impact of the T→S change at the ion coordinating residue at site 4 (Figure 8).

Although barium block of potassium channels has been very well characterized, susceptibility of potassium channels to amantadine is less well understood. Amantadine is better known as a blocker of the influenza proton channel M2 [18], [46], [47], but has been shown to block a number of different potassium channels [16], [48], [49]. In terms of physical-chemical properties, amantadine shares many features with quaternary amine compounds that have been widely used to explore potassium channel structure-function relationships [1], [50]. Most notably, amantadine is a hydrophobic amine. Quaternary amines have been shown to block potassium channels at an internal site that proximal to site 4 [51], [52]. Further, the interaction of many hydrophobic cation is thought to be facilitated by the hydrophobic nature of the lining of the internal cavity thought to be present in many potassium channels [53]–[56]. Amantadine readily partitions into lipid bilayers [57]–[59] and consequently could access the channel pore from the cytoplasmic side, even when the compound is applied externally. The discovery that a mutation on the internal side of the selectivity filter affects amantadine block suggests that amantadine blocks Kcv from the intracellular side. Thus, we conjecture that the probable mode of interaction would be for the primary amino group of the adamantyl ring to interact with site 4 while the remainder of the hydrophobic adamantyl cage interacts with hydrophobic residues from the M2 helices that are likely to line the channel pore. This binding mode would be similar to one of the ways amantadine has been shown to interact with the viral proton channel M2 [46].

Identifying and characterizing interactions between ion channels and blockers is an important area of research. The further development of genetic approaches such as those described here as well as novel technologies that facilitate the characterization of ion channel-blocker interactions [17] should provide new avenues for the discovery of ion channel modifiers for channels that presently lack pharmacologies.

## Methods

### Molecular Biology

PCR primers were used to amplify the gene for Kcv so that it carried EcoRI and XbaI sites at the 5′ and 3′ ends respectively. This PCR product was cloned into a yeast expression vector in which the channel gene is under control of the MET25 promoter [22] to make a vector called ‘pKcvY3’. Kcv P-stuffer is a non-functional clone bearing a 1 kb non-coding DNA fragment [22] cloned between the PvuII and HindIII sites of the gene.

### Library generation

An error-prone PCR procedure was used to generate random mutations in pKcvY3. Primers for the Met25 promoter and Cyc1Reverse primer that flank the entire gene were used for amplification by Taq polymerase in a buffer of 4 mM MgCl_2_, 1 mM MnCl_2_, 1 mM each of dCTP, dGTP, and dTTP, 0.2 mM dATP. 5′ 95°C, (95°C 45″,60°C 45″, 72°C 1′) for 40 cycles, followed by a 72°C 5′ extension step. Transformation of the library by cloning into the EcoRI and XbaI sites yielded a library of 2×10^4^. Sequencing of unselected clones showed a distribution with an average of 3.9 base changes (±2.3 s.d.) and 3.6 amino acid changes (±1.9 s.d.) that were distributed throughout the gene.

### Selection procedures

Yeast selections followed the protocols outlined in [22] and [9] with the following specialized changes. The library was plated on non-selective conditions 100 mM KCl (100 K) and grown at 30°C for two days. Following colony establishment, the library was replica-plated onto selective conditions: 1 mM KCl (1 K) or 0.5 mM KCl (0.5 K. Immediately following replica-plating 1 cm filter discs (Whatman part number 1823010) soaked with 100 µl of 100 mM or 10 mM BaCl_2_ or 500 mM amantadine were applied. Blocker-resistant colonies were chosen from colonies growing near the filter. Plasmid DNA was recovered and the phenotype was verified by retransformation and rescreening.

### PCR shuffling and backcrossing

A DNA shuffling approach was used following the general outline in [29], [60]. Genes for wild-type Kcv and Kcv* were amplified by PCR using primers to the parts of the plasmid that flanked the translated region of the gene. PCR products were purified (Qiagen), quantified by absorbance at 260 nm and mixed in a ratio of 1∶9 (Kcv*:Kcv) (900 ng∶8100 ng). The gene mixture at a total concentration of 23 ng/µl was digested by DNaseI (0.001 U/µl) in a buffer of 10 mM MgCl_2_, 50 mM Tris, pH 7.4 at 20°C for 2.5′. The reaction was stopped by incubation at 90°C for 10′. Digested products were run on an agarose gel. Gel fragments containing DNA fragments of size <220 bp were collected and electroeluted for 24 hours at 46 mV in 3500 mwco tubing in TAE. The solution containing the DNA fragments was phenol chloroform extracted, precipitated, and resuspended in 40 µl 1/10 TE. These fragments were subjected to two rounds of PCR using Pfu polymerase (Stratagene). In the first round, no primers were used. PCR was done using Pfu under conditions having 1 mM each of dCTP, dGTP, and dTTP, 0.2 mM dATP. 5′ 95°C, (94°C 1′, 65°C 1.5′, 59°C 1.5′, 56°C 1.5′, 53°C 1.5′, 50°C 1.5′, 47°C 1.5′, 44° C 1.5′, 41°C 1.5′, 72°C 5′) for 40 cycles, followed by a 72°C 10′ extension step. For the second PCR step, 1/10^th^ of the no-primer PCR reaction was used in a PCR reaction that included primers against the non-translated regions of the plasmid that flank the Kcv gene and that span the EcoRI and XhoI sites. The DNA from this step was phenol/chloroform extracted, ethanol precipitated, and resuspended in water and an aliquot was digested with XhoI and EcoRI. This fragment was gel purified and cloned into the a background in which a non-functional ‘stuffer sequence’ had been cloned between the XhoI and EcoRI sites to faciliate cloning [22]. The library was amplified and subjected to selection as outlined the ‘Selection procedures’ section.

### Membrane Preparation and Kcv stability assay

Kcv and Kcv T63S bearing a His_9_ tag at the N terminus were expressed in the methylotrophic yeast *Pichia pastoris* and membrane preparation was done according to Pagliuca *et al*. [15]. The resulting crude membrane pellets were suspended in ice-cold water, and the protein content was determined using the DC protein assay (Bio-Rad). For the stability assay, membranes containing 30 µg of protein were heated for 3 min at 95°C in Laemmli buffer (2% SDS, 10% glycerol, 100 mM DTT, 0.1% bromphenol blue, 62.5 mM Tris–HCl, pH 6.8) containing 200 mM of the indicated chloride salt and immediately after were placed in ice. Following protein separation by SDS-PAGE, Kcv protein was detected on Western blot probed with an anti-poly-His antibody (Sigma). Signal detection was performed with a second antibody coupled to alkaline phosphatase (Sigma) in the presence of commercial substrates (CDP-*Star* ^®^ Sigma).

### Electrophysiological measurements

Two-electrode voltage clamp experiments on Kcv were performed using a GeneClamp 500 amplifier (Axon Instruments) and digitized at 50 kHz with a Digidata 1200 (Axon Instruments). Data acquisition and analysis were done using the pCLAMP8 software package (Axon Instrument). Electrodes were filled with 3 M KCl and had resistances of 0.2–0.8 MΩ in 50 mM KCl. The oocytes were perfused at room temperature (25–27°C) with a bath solution containing: 50 mM KCl (or RbCl, CsCl, NaCl, or LiCl as indicated in the figure legend and text), 1.8 mM CaCl_2_, 1 mM MgCl_2_, 5 mM HEPES, adjusted to pH 7.4 with KOH (or RbOH, CsOH, NaOH, or LiOH) at a rate of 2 ml/min. Mannitol was used to adjust the osmolarity of the solution to 215 mosmol. BaCl_2_ was diluted from a 1 M stock and added to the various solutions as indicated. Amantadine was freshly prepared. The standard clamp protocol consisted of steps from the holding voltage of −20 mV to voltages in the range +80 mV to −160 mV. Tail currents were measured at −80 mV. Instantaneous and steady state currents were sampled after 10 ms and at the end of the voltage step (usually 800 ms), respectively. Two electrode voltage clamp recordings of Kir2.1 and Kir2.1 T142S were done using previously described methods [9].

Ion Permeability-Permeability ratios (PX/PK) were calculated according to the equation ΔE_rev_ = E_rev,X_−E_rev,K_ = RT/zF ln P_X_[X]_o_/P_K_[K]_o_. E_rev_ is the value in mV of the current reversal potential measured in the presence of the different monovalent cations in the external solution (either X^+^ or K^+^); [X]_o_ and [K]_ o_ are the cation concentrations in the external solution; and R, T, z and F have their usual thermodynamic meanings.

### Single channel analysis

Single channel recordings of Kir2.1 and Kir2.1 T142S were done using de-vitellinized oocytes following [9]. Single-channel currents were recorded by the patch-clamp technique in the cell-attached configuration as described previously [9], using an Axopatch 200B amplifier (Axon Instruments). The currents were digitized at 5 kHz using Digidata 1332A (Axon Instruments) and low-pass filtered at 1 kHz using an 8-pole Bessel filter (Frequency Devices). Pipette and Bath solution contained 45, 90, 150, or 300 mM KCl, 10 mM Hepes, 2 mM MgCl_2_, and 0.05 mM GdCl_2_ (pH 7.4). The 45 mM KCl solution contained in addition 45 mM NMDG. The 90 mM KCl solution is same as the TEVC recording solution. For dwell-time analysis, current records were analyzed using Clampfit 9.2. Single-channel transitions were detected by the 50% threshold crossing criterion. Events were log-binned at 10 bins per decade. The y ordinates of the histograms were square-root transformed for improved display of the multiple components [61]. Histograms were fitted using least-square minimization with the double or triple exponential components [62]. Bursts are defined by visual inspection, and the intraburst open probability was analyzed using Clampfit 9.2. Data presented is the mean±SD of the mean. Statistical significance was set at p<0.05 using t-test or one-way analysis of variance.
